# The role of microRNA-608 polymorphism on the susceptibility and survival of cancer: a meta-analysis

**DOI:** 10.18632/aging.101476

**Published:** 2018-06-16

**Authors:** Zhi-Ming Dai, Jian-Rui Lv, Kang Liu, Xiao-Ming Lei, Wei Li, Gang Wu, Xing-Han Liu, Yu-Xiao Zhu, Qian Hao, Zhi-Jun Dai

**Affiliations:** 1 Department of Anesthesiology, Second Affiliated Hospital of Xi’an Jiaotong University, Xi’an, Shaanxi 710004, China; 2 Department of Oncology, Second Affiliated Hospital of Xi’an Jiaotong University, Xi’an, Shaanxi 710004, China; * Equal contribution

**Keywords:** microRNA-608, polymorphism, cancer, susceptibility, prognosis

## Abstract

The role of rs4919510 polymorphism in microRNA-608 (miR-608) and cancer susceptibility and prognosis remain controversial and debatable. We conducted a meta-analysis of twenty-four eligible publications on the association of rs4919510 polymorphism with cancer risk and/or prognosis. Odds ratios, hazard ratios, and 95% confidence interval were used to investigate the association between this polymorphism and susceptibility, overall survival, and recurrence-free survival of cancer. Overall, eighteen case-control studies and nine cohort studies evaluated the susceptibility and prognostic value of rs4919510 polymorphism in cancer, respectively. Pooled analysis showed that rs4919510 polymorphism was not associated with cancer risk in all five genetic models. When stratifying by different cancer sites, rs4919510 polymorphism was detected to have a significant association with a decreased risk of colorectal cancer in homozygous model (*P* = 0.006) and recessive model (*P* = 0.001), subgroup analysis also emerged a weakened correlation between rs4919510 polymorphism and an increased risk of papillary thyroid cancer in heterozygote model (*P* = 0.04). Furthermore, the prognosis of rs4919510 variant in cancer patients showed that rs4919510 GG genotype was significant association with poor recurrence-free survival in homozygous models (*P* = 0.04). The meta-analysis suggested that the microRNA-608 rs4919510 polymorphism maybe associate with a significantly decreased risk for colorectal cancer. Further investigations on larger populations are required to evaluate and confirm this relationship.

## Introduction

MicroRNA (miRNA) is a growing family of naturally occurring, comprised of ~22 nucleotide, endogenous, single stranded non-coding RNA that is found in both prokaryotes and eukaryotes [1]. MiRNAs regulates the stability or translational efficiency of the targeted messenger RNAs (mRNAs) by targeting the 3' untranslated region (3´UTR) and altering the translation of genes at the post-transcription level [2]. At present, more than 1000 miRNA genes have been discovered in the human genome [3,4]. Numerous studies have indicated that miRNAs post-transcriptionally regulate a variety of biological processes, including cell proliferation, differentiation, apoptosis, and stress response. Thus, it is conceivable that the miRNA biogenesis pathway may have effects on cancer susceptibility, prognosis, and treatment response [5,6]. Single nucleotide polymorphisms (SNPs) are inherited genetic variations, which can affect gene expression or protein function. SNPs in miRNA genes may change the property of miRNAs by altering pri-miRNA/pre-miRNA processing, or by affecting the miRNA-mRNA interactions [7]. Therefore, SNPs in miRNAs may be considered as biomarkers for the diagnosis and/or prognosis of cancer.

The microRNA-608 (miR-608) gene lies within an intron of *SEMA4G* on the human chromosome 10q24 locus. Due to the loss of heterozygosity, the 10q24 locus has been reported to be correlated with certain types of human cancers, including brain, colorectal, prostate, and breast cancer [8-11]. The rs4919510 polymorphism, located in the mature miR-608 sequence, is a C/G single-nucleotide variation. The rs4919510 variation was considered to influence miRNA activities by binding to mir608 target sites within CD4 antigen, growth hormone receptor (*GHR*), retinoic X receptor beta (*RXRB*), and tumour protein p53 (*TP53*) genes with lower free energies than the wild type allele [12].

It has been hypothesised that rs4919510 polymorphism in miR-608 plays a part in carcinogenesis and clinic outcomes of cancer. Recently, several studies have revealed that the rs4919510 variation in the miR-608 gene could influence the susceptibility and/or prognosis of certain types of cancer, including colorectal [10,13-15], breast [11,16-19], thyroid [20,21], gastric [22,23], lung [24,25], head and neck cancers [26-28], and neuroblastoma [29], as well as esophageal squamous cell carcinoma [30], and hepatocellular carcinomas [31,32]. However, the results of the previous studies are inconsistent. In addition, an earlier meta-analysis found that rs4919510 polymorphism in miR-608 was significantly associated with decreased cancer risk in recessive model [33]. In recent years, there was a lot of new literature published. In this study, a phenotype meta-analysis was performed to evaluate the effects of miR-608 rs4919510 genetic polymorphism on susceptibility and prognosis of different types of cancer.

## RESULTS

### Characteristics of eligible studies

**Figure 1:**
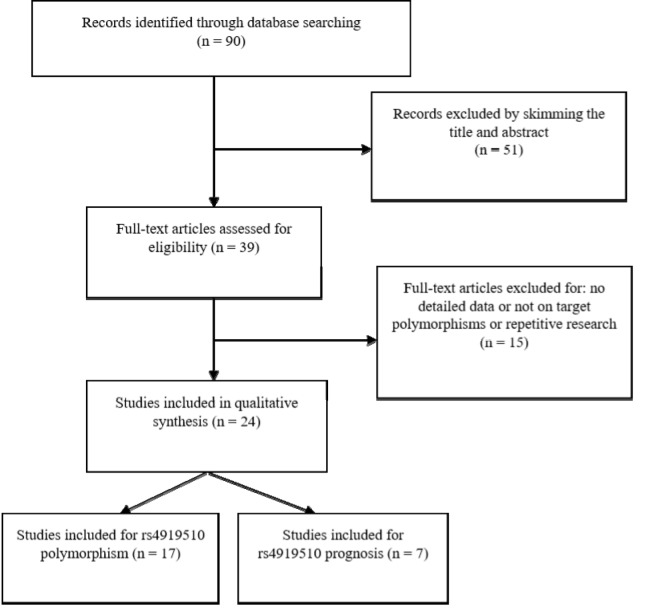
Preferred reporting items for systematic reviews and meta-analyses ﬂow diagram of the literature review process for microRNA-608 rs4919510 and cancer.

Through the primary literature retrieval in Pubmed, Embase, and CNKI, 90 studies were identified for cancer susceptibility and/or prognosis assessment based on rs4919510 polymorphism of miR-608 gene. After review of titles and abstracts, we identified 39 potential articles eligible to be included for the evaluation. After retrieving the literature, two overlapping studies were found [25,34], and we excluded the earlier study. Other two of the selected studies did not meet the requirements of Hardy-Weinberg Equilibrium (HWE) [35,36], and hence twenty-four articles were assessed for eligibility to be included in our meta-analysis. The flow chart for the studies selection process is shown in Fig. 1. There were two separate groups from different ethnicities (African-Americans and Caucasians) in Ryan et al’s study [10]. Thus, we treated them as separate studies. Another report by Zheng et al. [27] also included two independent studies of prognosis. Finally, eighteen independent case-control studies from seventeen published articles were evaluated for the association of rs4919510 polymorphism with cancer risk [10,11,13,15,17-23,25,26,28-31] and nine studies from seven published articles were evaluated for the prognostic value of rs4919510 polymorphism in cancer [10,14-16,24,27,32]. All eligible studies were published between 2012 and 2018 and carried out in the USA, China, Chile, Iran, and Lithuania. The studies presented data for several types of cancer including colorectal, gastric, lung, breast, thyroid, neuroblastoma and head and neck cancers as well as hepatocellular, and oesophageal squamous cell carcinomas. The characteristics of the eligible studies are presented in Table 1 and Table 2.

**Table 1:** Characteristics of studies evaluating cancer susceptibility included in the meta-analysis.

First author	Year	Country	Cancer type	Ethnicity	Study design	Genotyping method	Source of control	Case/Control	Cases			Controls			*P* of HWE	NOS
									GG	CG	CC	GG	CG	CC		
Ryan	2012	USA	CRC	AA	CC^*^	Taqman	Pop	94/185	12	48	34	28	95	62	0.39	9
				Caucasian	CC	Taqman	Pop	145/248	7	48	90	8	71	169	0.87	9
Huang	2012	China	BC	Asian	CC	SNPstream	Pop	1138/1934	381	545	192	640	914	354	0.38	8
Wang	2014	China	HCC	Asian	CC	MassArray	Pop	816/720	241	415	160	227	361	132	0.58	8
Kupcinskas1	2014	Lithuania	GC	Caucasian	CC	RT-PCR	Pop	363/350	25	88	250	13	86	251	0.11	8
Kupcinskas2	2014	Lithuania	CRC	Caucasian	CC	RT-PCR	Pop	192/426	7	47	138	12	96	318	0.16	7
Wei	2015	China	PTC	Asian	CC	MassArray	Pop	828/1031	266	428	130	326	503	202	0.75	8
Qiu	2015	China	NPC	Asian	CC	TaqMan	Pop	906/1072	255	460	191	254	532	286	0.83	8
Zhang	2015	China	ESCC	Asian	CC	SNaPshot	Pop	738/882	217	384	137	291	440	151	0.48	8
Dong	2015	China	PTC	Asian	CC	MassArray	Hosp	369/751	136	186	47	279	370	102	0.24	8
Jiang	2016	China	GC	Asian	CC	MassArray	Hosp	898/992	278	451	165	296	483	210	0.62	8
Miao	2016	China	HNC	Asian	CC	Illumina	Hosp	576/1552	177	285	114	509	762	278	0.81	8
Ying	2016	China	CRC	Asian	CC	MassArray	Pop	805/618	232	690	423	250	512	313	0.15	7
Dai	2016	China	BC	Asian	CC	MassArray	Pop	560/583	157	296	107	183	287	113	0.98	8
Hashemi	2016	Iran	BC	Caucasian	CC	PCR-RFLP	Pop	160/192	0	20	140	0	43	149	0.08	6
Morales	2016	Chile	BC	Caucasian	CC	Taqman	Pop	440/807	40	174	226	66	310	431	0.33	8
Yin	2016	China	LC	Asian	CC	TaqMan	Hosp	575/608	158	294	123	197	269	115	0.84	8
He	2018	China	Neuroblastoma	Asian	CC	Taqman	Pop	393/812	127	190	76	227	405	179	0.95	8

CRC: colorectal cancer;BC: breast cancer; HCC:hepatocellular carcinoma;GC: gastric cancer; PTC:papillary thyroid cancer;NPC: nasopharyngeal carcinoma; ESCC: esophageal squamous cell carcinoma; LC: lung cancer; HNC: head and neck cancer; AA: African-American; CC^*^: case-control Pop: population based; Hosp: hospital based; HWE: Hardy-Weinberg equilibrium; NOS Newcastle-Ottawa scale.

**Table 2:** Characteristics of studies evaluating cancer prognosis included in the meta-analysis.

Author	Country	Type of tumor	Ethnicity	Survival analysis	Mean/Median age (SD/range)	Stage	Cases	Genotypes	HR(95%CI)				Median follow-up (months)
OS (Univariable)	OS (Multivariable)	RFS (Univariable)	RFS (Multivariable)

Ryan 2012a	USA	CRC	AA	OS	64.7 (11.7)	I-IV	94	CG vs. CC	0.70 (0.39-1.27)	0.72 (0.40-1.30)	-	-	50.5
								GG vs. CC	0.38 (0.13-1.13)	0.36 (0.12-1.07)	-	-	
								CG/GG vs. CC	0.77 (0.53-1.12)	-	-	-	
Ryan 2012b	USA	CRC	Caucasian	OS	64.7 (11.7)	I-IV	145	CG vs. CC	1.25 (0.74-2.10)	1.41 (0.81-2.44)	-	-	50.5
								GG vs. CC	**3.54 (1.38-9.12)**	**2.95 (1.13-7.71)**	-	-	
								CG/GG vs. CC	1.24 (0.87-1.77)	**-**	-	-	
Xing 2012	China	CRC	Chinese	OS, RFS	59.4 (22-90)	0-IV	408	GC vs. CC	0.99 (0.60-1.64)	-	0.85 (0.57-1.27)	-	23
								GG vs. CC	1.11 (0.65-1.88)	-	1.21 (0.80-1.81)	-	
								CG/GG vs. CC	1.03 (0.64-1.66)	-	1.03(0.71-1.49)	-	
Zheng 2013a	China	NPC	Chinese	RFS	54 (21-82)	I-IV	873	CG vs. CC	-	-	1.41 (0.89-2.04)	1.45 (0.92-2.18)	37.2
								GG vs. CC	-	-	**2.02 (1.29-3.16)**	**2.05 (1.35-3.21)**	
								GC/GG vs. CC	-	-	**1.61 (1.13-2.29)**	**1.68 (1.18-2.61)**	
Zheng 2013b	China	NPC	Chinese	RFS	52 (19-87)	I-IV	828	CG vs. CC	-	-	**1.55 (1.03-2.15)**	**1.62 (1.11-2.19)**	34.8
								GG vs. CC	-	-	**2.21 (1.49-3.24)**	**2.24 (1.45-3.38)**	
								GC/GG vs. CC	-	-	**1.65 (1.17-2.39)**	**1.71 (1.23-2.31)**	
Jiao 2014	China	BC	Chinese	OS	51 (29-83)	I-III	196	CG vs. CC	1.42 (0.65-3.11)	-	-	-	114
								GG vs. CC	1.32 (0.72-2.41)	-	-	-	
								CG/GG vs. CC	1.34 (0.76-2.38)	-	-	-	
								CC/CG vs. GG	1.21 (0.61-2.40)	-	-	-	
Ma 2016	China	HCC	Chinese	OS	50 (20-81)	I-IV	361	GG vs. CC	**1.67 (1.11-2.49)**	**1.61 (1.07-2.40)**	-	-	53
								CG/GG vs. CC	**1.47 (1.03-2.09)**	1.40 (0.98-2.00)	-	-	
								CC/CG vs. GG	0.76 (0.57-1.01)	0.76 (0.57-1.01)	-	-	
Xia 2016	China	NSCLC	Chinese	OS	57.12 (11.47)	I-IV	584	CG vs. GG	1.07 (0.84-1.37)	0.98 (0.76-1.25)			-
								GG vs. CC	1.00 (0.74-1.36)	0.99 (0.73-1.34)			
Ying 2016	China	CRC	Chinese	OS, RFS	60.80 (13.03)	0-III	1218	CG vs. CC	0.89 (0.70-1.13)	0.89 (0.70-1.14)	0.92 (0.76-1.11)	0.93 (0.76-1.13)	26
								GG vs. CC	1.03 (0.75-1.41)	1.10 (0.80-1.52)	1.04 (0.81-1.34)	1.08 (0.83-1.40)	
								CG/GG vs. CC	0.92 (0.73-1.15)	0.94 (0.75-1.18)	0.94 (0.79-1.14)	0.97 (0.80-1.16)	
								CC/CG vs. GG	0.90 (0.68-1.19)	0.85 (0.64-1.12)	0.91 (0.72-1.15)	0.88 (0.70-1.10)	
								G vs. C	0.99 (0.85-1.16)	1.02 (0.87-1.19)	1.00 (0.89-1.13)	1.02 (0.90-1.16)	

CRC: colorectal cancer; NPC: nasopharyngeal carcinoma; BC: breast cancer; HCC:hepatocellular carcinoma; NSCLC: non-small cell lung cancer; AA: African-American; OS: overall survival; RFS: recurrence-free survival.

### The association between rs4919510 polymorphism and cancer susceptibility

**Figure 2:**
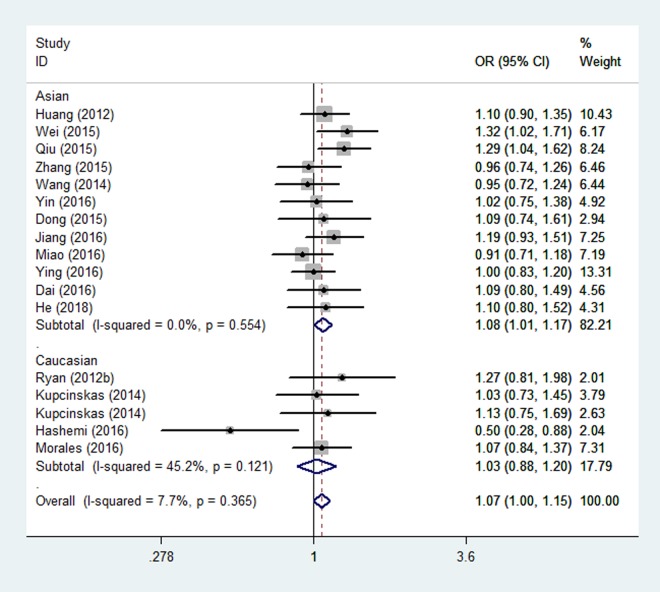
**Stratified analysis based on ethnicity for the association between microRNA-608 rs4919510 polymorphism and cancer risk using a heterozygous model (CG vs. CC).** The squares and horizontal lines correspond to the study specific OR and 95% CI. The area of the squares reﬂects the weight (inverse of the variance). The diamond represents the summary OR and 95% CI.

**Figure 3:**
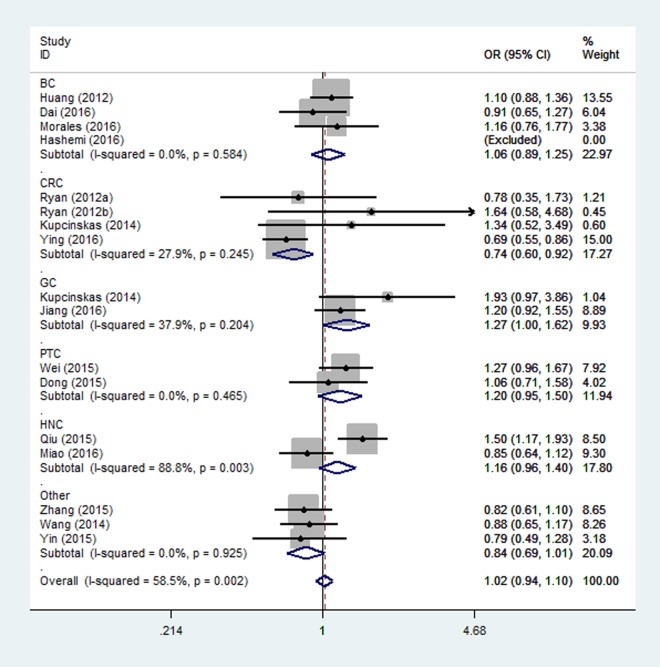
**Stratified analysis based on the diﬀerent cancer sites for the association between microRNA-608 rs4919510 polymorphism and cancer risk using homozygote model (GG vs. CC).** The squares and horizontal lines correspond to the study specific OR and 95% CI. The area of the squares reﬂects the weight (inverse of the variance). The diamond represents the summary OR and 95% CI.

Overall, there were eighteen studies encompassing 10,345 cases and 14,160 controls included in our meta-analysis. The associations of the rs4919510 polymorphism in miR-608 with the risk of different types of cancers are shown in Table 3. No statistically significant association was observed in the overall population under all comparison models. In the subgroup analysis of ethnicity, a significant association with increased cancer risk was found for Asians under heterozygote comparison (OR = 1.08, 95% CI = 1.01-1.17, Fig. 2). When the analysis was stratified by different cancer types, rs4919510 polymorphism was detected significant association with a decreased colorectal cancer risk in two genetic models (GG vs. CC: OR= 0.74, 95% CI = 0.60-0.92, Fig. 3; GG vs. CC/CG: OR= 0.73, 95% CI = 0.61-0.88). Moreover, significant associations were observed between rs4919510 polymorphism and papillary thyroid cancer in heterozygote model (OR = 1.25, 95% CI = 1.01-1.54).

**Table 3:** Meta-analysis results for the overall and subgroup analyses of rs4919510 polymorphism and cancer susceptibility.

Comparisons	G vs. C			GG vs. CC				GG vs. CC/CG			GG/CG vs. CC						CG vs. CC			
	OR (95% CI)	*P -value*	Heterogeneity (I^2^, *P-value*)	OR (95% CI)	*P -value*	Heterogeneity (I^2^, *P-value*)		OR (95% CI)	*P -value*	Heterogeneity (I^2^, *P-value*)	OR (95% CI)		*P -value*		Heterogeneity (I^2^, *P-value*)		OR (95% CI)		*P -value*	Heterogeneity (I^2^, *P-value*)	
**Overall**	1.01 (0.94-1.07)	0.84	62%, 0.001	1.03 (0.90-1.18)	0.64	61%, 0.001	0.97 (0.88-1.07)		0.54	56%, 0.003	1.05 (0.97-1.14)	0.25		39%, 0.05		1.07 (1.00-1.14)		0.05		4%, 0.41	
**Ethnicity**																					
Caucasian	1.08 (0.89-1.32)	0.45	51%, 0.09	1.36 (0.98-1.87)	0.06	0, 0.64	1.31 (0.96-1.80)		0.09	0, 0.61	1.05 (0.83-1.32)	0.69		50%, 0.09		1.03 (0.88-1.20)		0.75		45%, 0.12	
Asian	0.99 (0.93-1.07)	0.87	67%, 0.001	1.00 (0.86-1.16)	0.99	69%, 0.001	0.95 (0.86-1.05)		0.31	64%, 0.002	1.05 (0.96-1.16)	0.29		43%, 0.06		**1.08** **(1.01-1.17)**		**0.03**		0, 0.55	
**Tumor type**																					
BC	0.98 (0.85-1.13)	0.78	56%, 0.08	1.06 (0.89-1.25)	0.53	0, 0.58	0.99 (0.87-1.12)		0.84	0, 0.39	0.99 (0.80-1.22)	0.91		59%, 0.08		0.99 (0.80-1.24)		0.96		50%, 0.08	
CRC	0.99 (0.80-1.22)	0.91	50%, 0.08	**0.74** **(0.60-0.92)**	**0.006**	28%, 0.25	**0.73** **(0.61-0.88)**		**0.001**	21%, 0.28	0.97 (0.84-1.12)	0.64		16%, 0.32		1.04 (0.89-1.21)		0.64		0, 0.73	
GC	1.11 (0.99-1.25)	0.08	0, 0.40	1.27 (1.00-1.62)	0.05	38%, 0.20	1.11 (0.92-1.34)		0.29	63%, 0.10	1.18 (0.98-1.42)	0.09		0, 0.85		1.13 (0.93-1.38)		0.22		0, 0.50	
PTC	1.07 (0.96-1.19)	0.24	0, 0.49	1.20 (0.95-1.50)	0.12	0, 0.47	1.01 (0.87-1.19)		0.85	0, 0.80	1.23 (1.00-1.51)	0.05		0, 0.40		**1.25** **(1.01-1.54)**		**0.04**		0, 0.42	
HNC	1.06 (0.81-1.40)	0.66	89%, 0.003	1.13 (0.65-1.98)	0.66	0, 0.47	1.07 (0.77-1.48)		0.34	80%, 0.03	1.10 (0.72-1.68)	0.65		86%, 0.009		1.09 (0.78-1.54)		0.61		76%, 0.04	

OR: odds ratio; CI: confidence interval; BC: breast cancer; CRC: colorectal cancer; GC: gastric cancer; PTC: papillary thyroid cancer;HNC: head and neck cancer.

### The association between rs4919510 polymorphism and prognostic significance of cancer

Through retrieval in databases, nine studies reporting the association between rs4919510 polymorphism and the prognostic value of cancer were available. Survival data of the studies included our meta-analysis are summarised in Table 4. No significant association was observed between rs4919510 polymorphism and overall survival of cancer under univariate analysis or multivariate analysis. A significant result existing in homozygote comparison model indicates the GG allele maybe a significantly risk role in recurrence-free survival of cancer (GG vs CC: HR = 1.51, 95% CI = 1.02-2.23). However, the negative association was observed under multivariate analysis in homozygote model.

**Table 4:** Meta-analysis of the association between rs4919510 polymorphisms and prognosis of cancer.

Comparisons	Model	HR_Univariable_				HR_Multivariable_		
		HR (95% CI)		*p-value*		HR (95% CI)	*p-value*	
OS	GG vs. CC		1.20 (0.90-1.60)		0.22	1.18 (0.82-1.71)		0.38
	GG vs. CC/CG		1.05 (0.91-1.21)		0.51	1.12 (0.76-1.65)		0.57
	CG vs. CC		0.93 (0.79-1.09)		0.36	0.92 (0.75-1.14)		0.45
RFS	GG vs. CC		1.51 (1.02-2.23)		**0.04**	1.67 (1.01-2.76)		0.05
	GG vs. CC/CG		1.24 (0.91-1.70)		0.17	1.38 (0.91-2.09)		0.13
	CG vs. CC		1.10 (0.83-1.47)		0.49	1.26 (0.85-1.86)		0.25

OS: overall survival; RFS: recurrence-free survival; HR: hazard ratio; CI: confidential interval; HR_Multivariable_, HR adjusted by gender, age, histological type, clinical stage, receipt of chemotherapy.

### Publication bias

**Figure 4:**
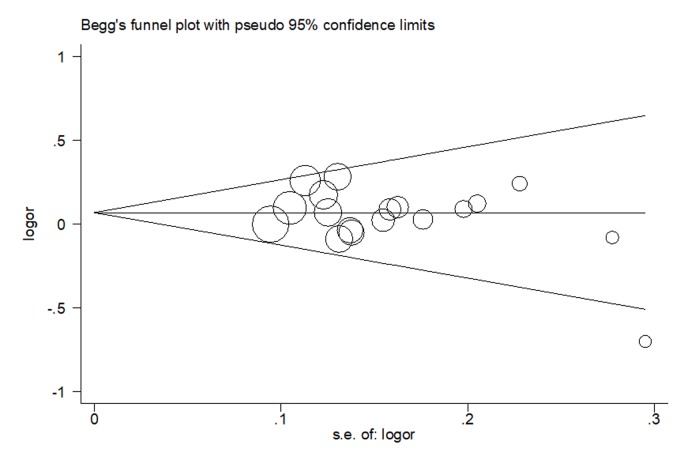
Begg’s funnel plot of publication bias on the relationships between rs4919510 polymorphism and cancer susceptibility under heterozygous model.

In our meta-analysis, both Begg’s funnel plot and Egger’s test were conducted to assess the publication bias. As shown in Fig. 4, the results revealed no publication bias for eligible studies under heterozygous model. The symmetry was then confirmed in Egger’s linear regression test (*P* = 0.21).

### Heterogeneity and sensitivity analysis

**Figure 5:**
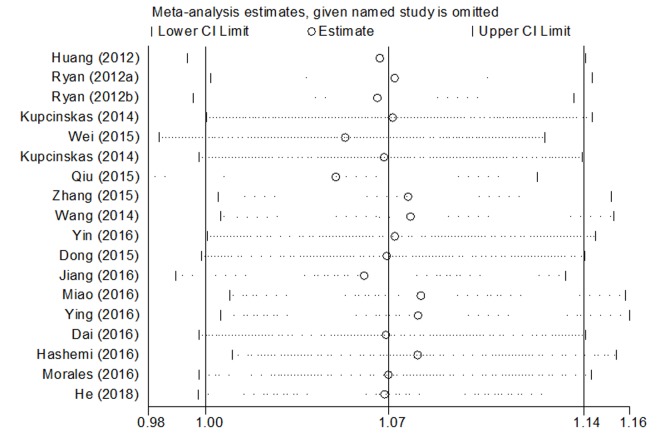
Sensitivity analysis of association between microRNA-608 rs4919510 polymorphism and cancer risk.

Significant heterogeneities in the data of microRNA-608 rs4919510 polymorphism were observed in the overall meta-analysis as well as subgroup analysis, the results are shown in Table 3. Sources of heterogeneity were further studied by sensitivity analysis, the result showed that no individual study influenced the pooled OR value (Fig. 5), indicating that the results of our meta-analysis are statistically credible and robust.

## DISCUSSION

Aberrant miRNA function and expression could influence multiple physiological processes and participate in the occurrence, development, and prognosis of many human diseases including malignant tumours [7,37]. Furthermore, SNPs or mutations in miRNAs may directly exert their effects by binding to target mRNAs [38]. The rs4919510 polymorphism was located in the sequence of mature miR-608 and its G/C variation was common in several populations. A previous report found that the variant miR-608 was predicted to bind to mir608 target sites within immunity and defense, DNA repair, cell growth-related, and cell death-related genes with lower free energies than that of the wild type allele, for example, CD4, GHR, RXRB, and TP53 [12]. Therefore, it was assumed that the rs4919510 polymorphism possibly elicits different biological activities of these target mRNAs and is associated with the pathogenesis and prognosis of cancer as a predictive biomarker.

Our meta-analysis results showed that no significant association was observed between rs4919510 variant in miR-608 and risk of cancer in the overall population based on seventeen eligible case-control studies. However, an association was found between rs4919510 polymorphism and cancer risk in an Asian population based on the heterozygote model (*P* = 0.03), meanwhile, similar positive results were observed in PTC patient (*P* = 0.04). Therefore, rs4919510 polymorphism might be potential indicators of PTC risk.

A previous study found that rs4919510 polymorphism was not significantly associated with BC risk, but variant genotypes (GG/CG) influenced HER2-positive BC risk [11]. The results our own group proved rs4919510 alleles of miR-608 were not associated with the presence of BC a Northern Chinese population [17]. Negative results have also been reported in Chile populations [19]. Conversely, another research results revealed that rs4919510 GC genotype significantly decreased the risk of BC compared to CC genotype in an Iran population [18]. In a published pooled analysis, Wang et al [39] reported that there is no relationship between rs4919510 polymorphism and BC risk, our findings were partially in line with results from this meta-analysis.

Ryan et al. [10] and Kupcinskashas et al.’s [13] studies indicated no significant associations between rs4919510 polymorphism and CRC risk in Caucasian and African American populations. Opposite results were proved by Ying et al. who found that rs4919510 GG genotype was significantly associated with a decreased susceptibility to colorectal cancer in homozygous and recessive genetic models [15]. Remarkably, our meta-analysis results showed that rs4919510 GG genotype was significantly associated with a decreased susceptibility to CRC in homozygous and recessive genetic models. The results suggested that the presence of GG models might play a protect role in tumorigenesis in colorectal cancer. The strength of his conclusion should be confirmed on the base of more studies and sample size.

Qiu et al. [26] showed that carriers of rs4919510 CC genotypes had a reduced risk of nasopharyngeal carcinoma versus individuals who carried GG/CG genotypes in Chinese population. However, negative results were also reported in Chinese populations by Miao et al. [28]. Moreover, null significant association between this polymorphism and gastric cancer risk in Kupcinskas et al. [22] and Jiang et al.’s [23] studies. The other studies by Wang et al. [31] and Zhang et al. [30] showed that rs4919510 polymorphism was not associated with HCC and ESCC susceptibility respectively.

To date, there are three meta-analyses concerning the correlation of rs4919510 genetic susceptibility to cancer. Hu et al.’s study [40] indicated that no significant associations between rs4919510 polymorphism and overall risk of cancer or the risk of specific types of cancer. Another pooled analysis observed that rs4919510 polymorphism was significantly associated with elevated cancer risk in Chinese [41], however, the meta-analysis included some published article that the control population was not conformed the requirements of Hardy-Weinberg Equilibrium. Liu et al. [33] showed that rs4919510 variant was significantly associated with decreased cancer risk based on recessive model. Our study included more case-control studies than those two previous researches and the results challenged of their findings and provided stronger evidence.

Polymorphism of rs4919510 in miR-608 has been little explored for its effect on clinical outcomes for different types of cancer. Lin et al. [42] found that rs4919510 variation was associated with an increased renal cell carcinoma risk. Xing et al. [14] reported that the rs4919510 C allele was associated with a reduced risk of colorectal cancer and had a greater RFS and OS in a Chinese population. Interestingly, a study on colorectal cancer by Ryan et al. [10] showed that the rs4919510 GG genotype was associated with an increased risk of death in Caucasians and with a reduced risk of death in African Americans. However, negative results were reported in Asian populations in different types of cancer [15,16,24]. Furthermore, Zheng et al. [27] found that rs4919510 polymorphism was not associated with RFS but with OS of nasopharyngeal carcinoma in Asians.

As far as we know, this is the first meta-analysis regarding the rs4919510 polymorphism in miR-608 and its clinical prognosis significance in cancer. The results indicated that rs4919510 variant was significantly associated with the RFS of cancer under homozygote models (*P* = 0.04) in Asians by univariable analysis, however, which was no longer remained significant after adjustment for age, gender, histology and clinic stage and so on in multivariable model. No significant association was observed between this polymorphism and the OS of cancer. This might result from the different types of cancers, differences in clinical trials including different ethical backgrounds, different baseline characteristics, or various follow-up time periods used in each study. Furthermore, the exact functional mechanism by which rs4919510 polymorphism in miR-608 may affect cancer prognosis, there are several plausible hypotheses such as the polymorphism altering the transcription of target genes and reducing the sensitivity of the cancer cell to chemotherapeutic drugs [7,43].

Our study provides added evidence that the rs4919510 gene polymorphism may be associated with cancer susceptibility and prognosis. However, some potential limitations of our meta-analysis should be acknowledged. First, the sample size was relatively small in some studies, which may reduce the statistical power of the analysis and needs to be interpreted with caution. Second, in our meta-analysis, some cancers only had one study included, such as hepatocellular carcinoma, esophageal squamous cell carcinoma, lung cancer and neuroblastoma, which may lead to heterogeneity in quantitative analysis. Third, we perform stratified analysis by population, most of which are Chinese, and type of cancer, most of which are BC and CRC, rather than other baseline characteristics. Fourth, there was significant heterogeneity in some comparison models in our meta-analysis. Even with the use the appropriate meta-analytical techniques with the random-effects model, the heterogeneity might affect the precision of the overall results.

In conclusion, this meta-analysis suggests that the rs4919510 polymorphism in miR-608 is associated with cancer risk and prognosis. Further well-designed, prospective investigations including rs4919510 polymorphism and cancer susceptibility and prognosis with larger sample sizes are required in order to validate our findings.

## METHODS

### Literature search and selection criteria

This meta-analysis followed the Preferred Reporting Items for Systematic Reviews and Meta-analyses (PRISMA) criteria [44]. We searched PubMed, Embase, and Chinese National Knowledge Infrastructure (CNKI) databases for studies published prior to March 2018 (last search: March 30, 2018). The search was conducted with and without medical subject heading (MeSH) terms for ‘‘MicroRNA-608/rs4919510’’, ‘‘polymorphism or variant or mutation or genotype’’, and ‘‘cancer’’. The languages were limited to English and the subjects were human. The reference lists of searched literatures were read manually to complete our investigation, if necessary. It is necessary to select appraisal quality tools to evaluate the selected literature [45]. The Newcastle-Ottawa Scale (NOS) was used for assessing the quality of case-control study, the results were showed in Table 1. The NOS score of all articles are not less than 6 scores that mean each selected literature was a high-quality study.

Studies that fit into our meta-analysis fell into one of the following categories: (1) evaluation of the association of rs4919510 polymorphism and cancer susceptibility and/or prognosis; (2) provision of sufficient data to estimate the odds ratio (OR) or hazard ratio (HR) and 95% confidence intervals (CI) according to rs4919510 polymorphism; (3) case-control design; (4) controls without any evidence of malignant disease. The following were the exclusion criteria: (1) repeats studies, reviews, and abstracts; (2) the study did not have a control group; (3) design based on family; (4) the control population was not conformed the requirements of Hardy-Weinberg Equilibrium. The diagnosis of each cancer in eligible study was made based on the histopathological evidence and those with secondary tumour or metastasized cancer from other organs were excluded. Two investigators (Z-MD and J-RL) retrieved the literature and extracted data from each eligible study independently.

### Data extraction and synthesis

For each eligible study investigating the association between rs4919510 polymorphism and cancer risk, the following information was extracted by the two independent researchers: first author, year of publication, country, tumour type, ethnicity, source of control, size of the study population, genotyping methods, as well as Hardy-Weinberg value of controls. For studies examining the association between rs4919510 polymorphism and its prognostic value in cancer, the following information was collected from the eligible articles: first author, year of publication, origin of the study population, type of cancer, method of survival analysis, HRs with 95% CIs, as well as the follow-up time. Different ethnic groups were categorised as Caucasian, Asian, and African. Disagreements were resolved by discussion among all authors.

### Statistical analysis

ORs and 95% CIs were used to measure cancer risk associated with the rs4919510 polymorphism. Five different ORs were calculated: (1) GG versus CC (homozygous carriers), (2) CG versus CC (heterozygous carriers), (3) GG+ CG versus CC (dominant model), (4) GG versus CC + CG (recessive model), and (5) G versus C (allele comparison). The Hardy-Weinberg equilibrium (HWE) for the controls was assessed by the Chi-square test in each study. HRs with their 95% CIs was combined to evaluate the effective value of the rs4919510 polymorphism on the prognosis of cancer. The significance of the pooled ORs/HRs was determined by the Z test. If the study did not report the HRs, the Engauge Digitizer version 4.1 was used to read the Kaplane-Meier curves to estimate the HRs and the 95% CIs.

Statistical heterogeneity between studies was measured by the Chi-square-based Q statistic and evaluated with the I^2^ test; higher I^2^ values means higher levels of heterogeneity (I^2^ = 75-100%: extreme heterogeneity; I^2^ = 50-75%: large heterogeneity; I^2^ = 25-50%: moderate heterogeneity; I^2^ < 25%: no heterogeneity) [46]. When the *p* value was greater than 0.10, the fixed-effects model was used, otherwise the random-effects model was used. The Egger’s test and Begg’s funnel plot were adopted to assess publication bias. Sensitivity analysis determined whether the individual data in fact have a major effect on the results of the review. All analysis was performed by the Review Manager version 5.3 (The Cochrane Collaboration, Oxford, United Kingdom) and the Stata version 14.0 (Stata Corp, College Station, TX).
